# Effects of Balance Exercise Assist Robot training for patients with hemiparetic stroke: a randomized controlled trial

**DOI:** 10.1186/s12984-022-00989-6

**Published:** 2022-01-28

**Authors:** Seigo Inoue, Yohei Otaka, Masashi Kumagai, Masafumi Sugasawa, Naoki Mori, Kunitsugu Kondo

**Affiliations:** 1 Department of Rehabilitation Medicine, Tokyo Bay Rehabilitation Hospital, Chiba, Japan; 2 Department of Rehabilitation Medicine I, School of Medicine, Fujita Health University, 1-98 Dengakugakubo, Kutsukake, Toyoake, Aichi 470-1192 Japan

**Keywords:** Cerebrovascular disorder, Exercise, Robotics, Physical therapy, Postural balance, Rehabilitation

## Abstract

**Background:**

Robot-assisted rehabilitation for patients with stroke is promising. However, it is unclear whether additional balance training using a balance-focused robot combined with conventional rehabilitation programs supplements the balance function in patients with stroke. The purpose of this study was to compare the effects of Balance Exercise Assist Robot (BEAR) training combined with conventional inpatient rehabilitation training to those of conventional inpatient rehabilitation only in patients with hemiparetic stroke. We also aimed to determine whether BEAR training was superior to intensive balance training.

**Methods:**

This assessor-blinded randomized controlled trial included 60 patients with first-ever hemiparetic stroke, admitted to rehabilitation wards between December 2016 and February 2019. Patients were randomly assigned to one of three groups, robotic balance training and conventional inpatient rehabilitation (BEAR group), intensive balance training and conventional inpatient rehabilitation (IBT group), or conventional inpatient rehabilitation-only (CR group). The intervention duration was 2 weeks, with assessments conducted pre- and post-intervention, and at 2 weeks follow-up. The primary outcome measure was a change in the Mini-Balance Evaluation Systems Test (Mini-BESTest) score from baseline.

**Results:**

In total, 57 patients completed the intervention, and 48 patients were evaluated at the follow-up. Significant improvements in Mini-BESTest score were observed in the BEAR and IBT groups compared with in the CR group post-intervention and after the 2-week follow-up period (P < 0.05).

**Conclusions:**

The addition of balance exercises using the BEAR alongside conventional inpatient rehabilitation improved balance in patients with subacute stroke.

***Trial registration*:**

https://www.umin.ac.jp/ctr; Unique Identifier: UMIN000025129. Registered on 2 December 2016.

## Background

Balance can be defined as the ability to maintain and restore the center of gravity line when the base of support continuously changes [1]. Balance control involves different underlying systems, including anticipatory postural adjustments, postural responses, sensory orientation, and balance during gait [2]. Balance issues are frequently observed in patients with stroke and are closely related to mobility [3] and an increased risk of falling [4]. Among the various types of balance rehabilitation for patients with stroke [5], robot technology has gained attention as a potentially more efficient intervention. Importantly, repetition of task-specific activities for patients with stroke is effective in improving functional ability [6]. In this context, robots are considered to have great potential because of their strength in facilitating repetitive tasks. As a form of robotic intervention, robot-assisted gait training has been widely known and reported to improve walking ability [7] and balance [8, 9]. Considering task specificity, the use of a robots specialized in balance training is desirable; however, few studies assessing the usefulness of robot-assisted training, specifically focused on balance, have been undertaken. Notably, the Balance Exercise Assist Robot (BEAR, TOYOTA Motor Corporation, Aichi, Japan) is specialized in balance training [10]. The BEAR is a stand-up robot integrated with a video game that uses information such as velocity and body gradients obtained from a sensing device to adjust the training regime, and is classified as a surface-, mobile-, or platform-type robot [11]. Studies using the BEAR for patients with central nervous system disorders [10] and older adults with frailty [12] have reported improvements in dynamic balance ability and lower extremity muscle strength after training. However, to the best of our knowledge, the effectiveness of BEAR training compared with that of conventional balance training for patients with stroke has not been investigated.

Reportedly, balance training, including reaching movements and weight shifting, adjustment of motor responses to changes in body movements, and strengthening of lower limb muscle strength, is an important form of exercise therapy for balance improvement in patients with stroke [13, 14]. However, importantly, it is unclear whether additional balance training in combination with conventional rehabilitation programs supplements the balance function in patients with stroke [15]. Although a recent meta-analysis that included studies with homogeneous clinical outcomes [16] found a positive effect of additional balance exercises on balance function in patients with stroke, mixed results prevent confirmation of the efficacy of additional balance training. For example, while several randomized controlled trials found that additional balance exercises had no effect on balance function [17–19], other randomized controlled trials [20–23] reported the positive effects of additional training on balance function in patients with stroke. Furthermore, no study has examined the effectiveness of additional balance training on balance function using a balance-focused robot.

Therefore, we aimed to determine the effect of BEAR training on balance in combination with conventional inpatient rehabilitation training compared to the effects of conventional inpatient rehabilitation alone in patients with hemiparetic stroke. Moreover, we aimed to determine whether BEAR training was superior to dose-matched supervised intensive balance training.

## Methods

### Trial design

This trial was designed as an assessor-blinded, randomized controlled trial based on the CONSORT statement. The study protocol was approved by the Institutional Review Board of Tokyo Bay Rehabilitation Hospital, Japan (approval number 145–5), which was registered before the study (UMIN000025129). This study was conducted as per the Declaration of Helsinki (revised in 2013), and all patients provided written informed consent before study enrollment.

### Study setting and participants

The study was conducted at the Tokyo Bay Rehabilitation Hospital, which has convalescent rehabilitation wards [24]. All patients with stroke who were admitted to the hospital between December 2016 and February 2019 were consecutively screened, and one of the authors obtained informed consent from the patients for participation in this study. The inclusion criteria for this study were as follows: age, 40–80 years; first-ever hemiparetic stroke in the early subacute phase (1 week to 3 months after stroke onset) [25]; weight, 35–100 kg; height, 140–190 cm; no apparent paralysis in the unaffected limbs; no severe contractures and deformity in the lower limbs; able to stand still on the BEAR; and Functional Ambulation Category score ≥ 2 [26]. Exclusion criteria included: patients unable to follow instructions due to aphasia and/or cognitive deficits; patients with medical conditions that deteriorate with exercise; patients with preserved balance function and a Mini-Balance Evaluation Systems test (Mini-BESTest) score of ≥ 25 [27]; and patients assessed as unsuitable for participation in the study by the physician in charge.

### Intervention

Patients were randomly assigned to one of the following three groups: the robotic balance training combined with conventional inpatient rehabilitation (BEAR group), intensive balance training (IBT) combined with conventional rehabilitation (IBT group), or conventional inpatient rehabilitation-only (CR group) groups. The study duration was 4 weeks, comprising a 2-week intervention period and a 2-week follow-up period. For all three groups, conventional inpatient rehabilitation programs, including 60 min of physical, 60 min of occupational, and speech-language therapy if indicated, were provided for up to 180 min per day. Moreover, patients in the BEAR and IBT groups underwent an additional 18 min training session six times a week during the intervention period.

**Fig. 1:**
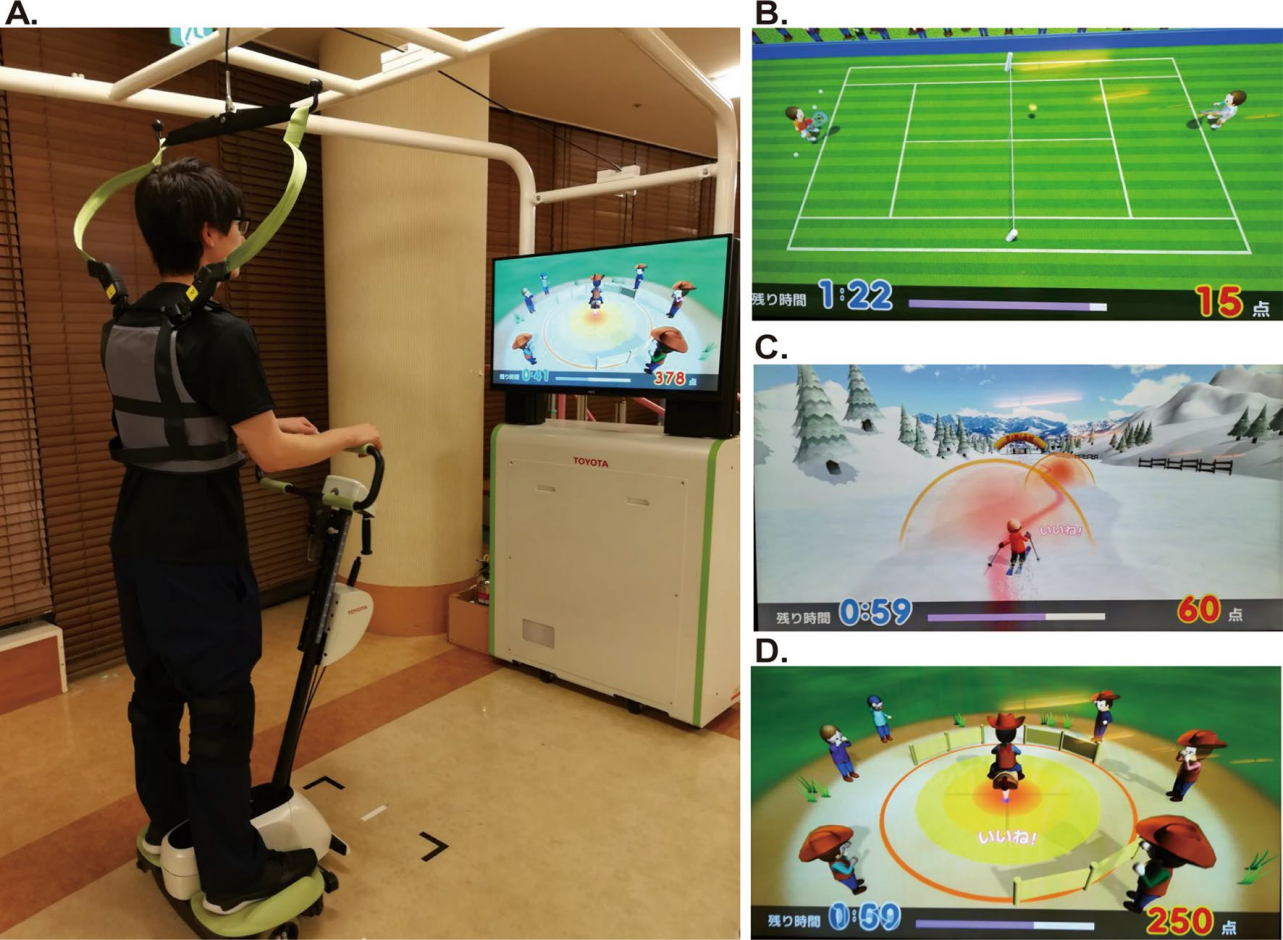
Balance Exercise Assist Robot (BEAR). **A** Overview. **B** Tennis game with active forward and backward center of gravity movement. **C** Ski game with active left–right weight shift. **D** Rodeo game in which the patient is required to keep the robot stationary against irregular disturbances

**Fig. 2:**
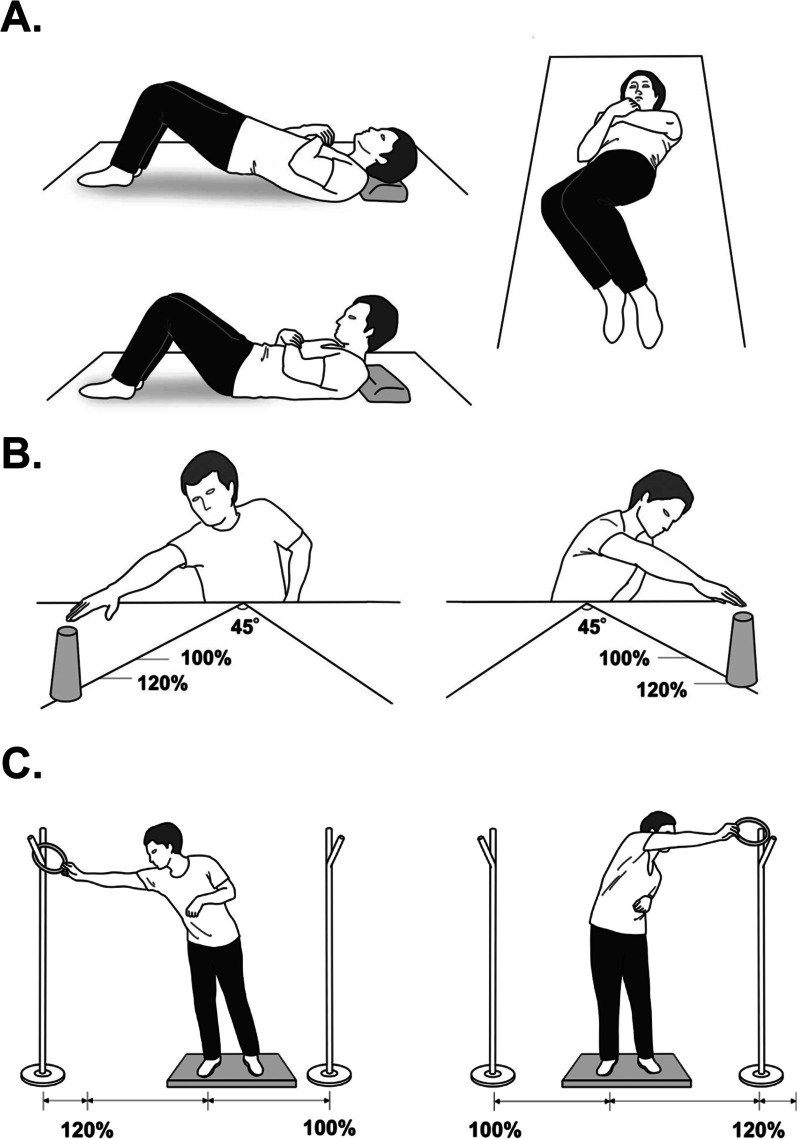
Intensive balance training. **A** Trunk training in the supine position. **B** Dynamic balance training performed in a seated position. **C** Dynamic balance training performed in a standing position

The BEAR is a standing robot integrated with a video game and is a system specialized for balance training, consisting of a robot, a monitor, and a safety hanging device (Fig. 1A). Participants boarded the BEAR and performed the following three balance tasks for 18 min: (1) anticipatory and reactive postural control, namely, tennis, with active forward and backward movement of the center of gravity (90 s × 4 times, Fig. 1B); (2) skiing, with active side-to-side movement of the center of gravity (90 s × 4 times, Fig. 1C); and (3) rodeo, keeping the robot stationary against irregular disturbances (90 s × 4 times, Fig. 1D). The robot automatically changed the difficulty level of the balancing task (game) according to the level of achievement. In the present study, a physical therapist who had mastered the operation of BEAR watched over the training just in case.

For the IBT group, we developed a supervised balance training program that included training components demonstrating positive effects when combined with the conventional rehabilitation program in previous studies [20, 21]. The 18-min training session consisted of core muscle strengthening, performed in the supine position; dynamic balance, performed in the seated and standing positions (Fig. 2); and trunk flexion, extension, and shaking in a supine position for 120 s in total (two sets, 20 s each; Fig. 2A). While seated, patients used the non-affected arm to reach right and left targets that had been placed on a table at an angle of 45º for 480 s in total (two sets, 60 s each with and without an air cushion; Fig. 2B). Patients used their non-affected hand when standing to reach and place a small hoop over a pole at a set distance on the left and right sides for a total of 480 s (two sets, 60 s each with and without a foam mat; Fig. 2C). For reach training, while seated and standing (Fig. 2B and C), the range of each reach was set to 120% of a patient’s arm length, and the distance was extended a further 10% when a patient was able to reach the target. When a patient was unable to reach the target, we set the target at the previous position and then extended it 1% further, so that the patient could perform the task under the maximum difficulty level.

### Outcomes

Assessments were conducted pre- and post-intervention and at the end of the 2-week follow-up period; the assessment took approximately one hour each. The assessors were physical therapists who were not involved in the study, had a clinical experience of > 6 years, and were well-trained in the assessments used in the study.

The primary outcome measure was the Mini-BESTest, a 14-item clinical test that covers four components of dynamic balance (anticipatory postural adjustments, postural responses, sensory orientation, and stability in gait) [27]. Each item is scored from 0 (unable or requiring help) to 2 (normal), with a maximum score of 28 points. A greater score indicates better balance. This test has had excellent intra-examiner reliability and validity [28, 29] and its advantage is its small ceiling effect in patients with stroke [28, 29]. The reliability of the scores for the four dynamic balance subsystems has also been confirmed [28].

Further, we assessed the following secondary outcomes:

The motor domain of Stroke Impairment Assessment Set was assessed as an index of motor function. The measure is a comprehensive measure of impairments in patients with stroke [30], whose reliability and validity have been previously confirmed [30, 31]. Motor scores consist of two tests for the upper extremity (0–10) and three tests for the lower extremity (0–15). To assess the severity of lower limb paralysis, we employed the sum of the lower limb motor item scores.

We utilized the Functional Independence Measure as an index for the activities of daily living. Items of the index are scored using a 7-point scale, where 1 indicates complete dependence and 7 indicates complete independence; the index comprises 13 motor subscales (13–91 points) and 5 cognitive subscales (5–35 points) [32, 33]. The reliability and validity of this measure have been previously confirmed in patients with stroke [34].

We employed the Functional Ambulation Category as an index of walking ability. It is scored on a scale of 0–5, with higher scores indicating higher walking independence [26]. The reliability and validity of the Functional Ambulation Category have been previously confirmed in patients with stroke [35].

The Timed Up and Go test was used as another metric of dynamic balance measure. It records the time taken to rise from an armchair, walk 3 m, turn, and return to a seated position [36]. The reliability and validity of this instrument have been confirmed in patients with stroke [37].

Based on a previous study [38], the maximal movements of the center of pressure from front to back and left to right were measured in an open-eyed standing position with their feet a shoulder-width apart on a force plate (Kinetogravicorder G-7100, Anima, Tokyo, Japan).

Bilateral hip flexion/abduction, knee flexion/extension, and ankle dorsiflexion/plantarflexion strength (N) were measured using a handheld dynamometer (PowerTrackII, Nihon Medix, Tokyo, Japan). The position of the joint in each measurement was set to be an isometric contraction using a belt [39]. The reliability of lower limb isometric muscle strength measurement using a hand-held dynamometer has been previously confirmed in patients with stroke [40].

Peak bilateral lower limb extension torque was measured during isokinetic cyclic movement using a recumbent cycle ergometer at 20 rpm (Strength Ergo 240™; Mitsubishi Electric Corporation, Tokyo, Japan). This test has shown excellent test–retest reliability and sufficient validity against a conventional isokinetic dynamometer [41]. Torque measures divided by body weight (Nm/kg) were used for the analyses.

The Fall Efficacy Scale-International was used to assess fear of falling [42]. In the evaluation method, patients are interviewed regarding their anxiety about falling while performing 16 activities; this anxiety is scored on a 4-point Likert scale (1, not at all concerned; 4, very concerned; 16–64 points), with higher scores indicating a greater fear of falling [42]. The reliability and validity of this instrument have also been previously confirmed [43].

Using a training questionnaire, based on a visual analog scale, patients were asked to slash check how they felt about their training on a 100-mm line. They were asked the following three questions: 1) "Did you feel that this exercise was effective?” (effectiveness); 2) "Did you enjoy this exercise?” (enjoyment); and 3) "Did you feel like you wanted to continue doing this?” (adherence). We adopted the usual walking exercise as a reference and the center of the line at 50 mm from the line edges. Walking is the most frequently performed movement in physical therapy [44] and was set as the representative value criterion for conventional training.

Any falls that occurred during the study were identified from patient incident reports, and unintended phenomena, symptoms, or illnesses that occurred were considered adverse events.

### Sample size

The primary aim of the study was determine whether the results from the BEAR group were superior to those from the CR group in terms of changes from baseline values in the Mini-BESTest. To estimate the sample size, the minimal clinically important difference in the total Mini-BESTest score in patients with stroke (i.e., 4 points) [29] was used to set the detected difference between the BEAR and CR groups. Using a standard deviation of 3.9 for the change in the total Mini-BESTest score, based on a previous study involving an inpatient rehabilitation program in patients with subacute stroke [45], we calculated an effect size as 1.025, with a significance level of 0.05 and a power (1-β) of 0.8. Based on the Student’s t-test, a total of 16 patients in each group was required for meaningful analyses. Considering some dropouts and unavailability of data, the number of patients was set at 20 in each group, for a total of 60 patients.

### Randomization

Using computer-generated random numbers, patients were randomly assigned in blocks to one of the three groups (block size, 6). The allocation process was performed by a person not involved in this study, and concealment was retained until allocation completion. All assessors were blinded to the patient assignment throughout the study.

### Statistical method

Baseline variables were compared between groups using one-way analysis of variance (ANOVA) and Kruskal–Wallis or Fisher’s exact tests, depending on the variables’ characteristics. For all outcome measures, a modified intention-to-treat analysis was performed. For between-group comparisons, differences from baseline values after intervention and at follow-up were compared using analysis of covariance (ANCOVA). Considering that age and sex differed significantly among the groups at baseline along with possible confounders, we used these variables and baseline values for each variable as covariates for adjustment. When the ANCOVA indicated a significant difference among groups, we performed post-hoc pairwise comparisons using the Bonferroni correction. Regarding within-group comparisons, values at baseline, post-intervention, and follow-up were compared using repeated measures ANOVA tests with post-hoc pairwise comparisons using the Bonferroni correction. The Mann–Whitney test was performed to compare the results from the questionnaires administered post-intervention between the BEAR and IBT groups. Negative binomial regression was performed to analyze fall counts between the groups. Data analyses were performed using STATA/MP 15.1 (StataCorp., Texas, USA). *P*-values of < 0.05 were considered statistically significant.

## Results

### Participant flow

**Fig. 3:**
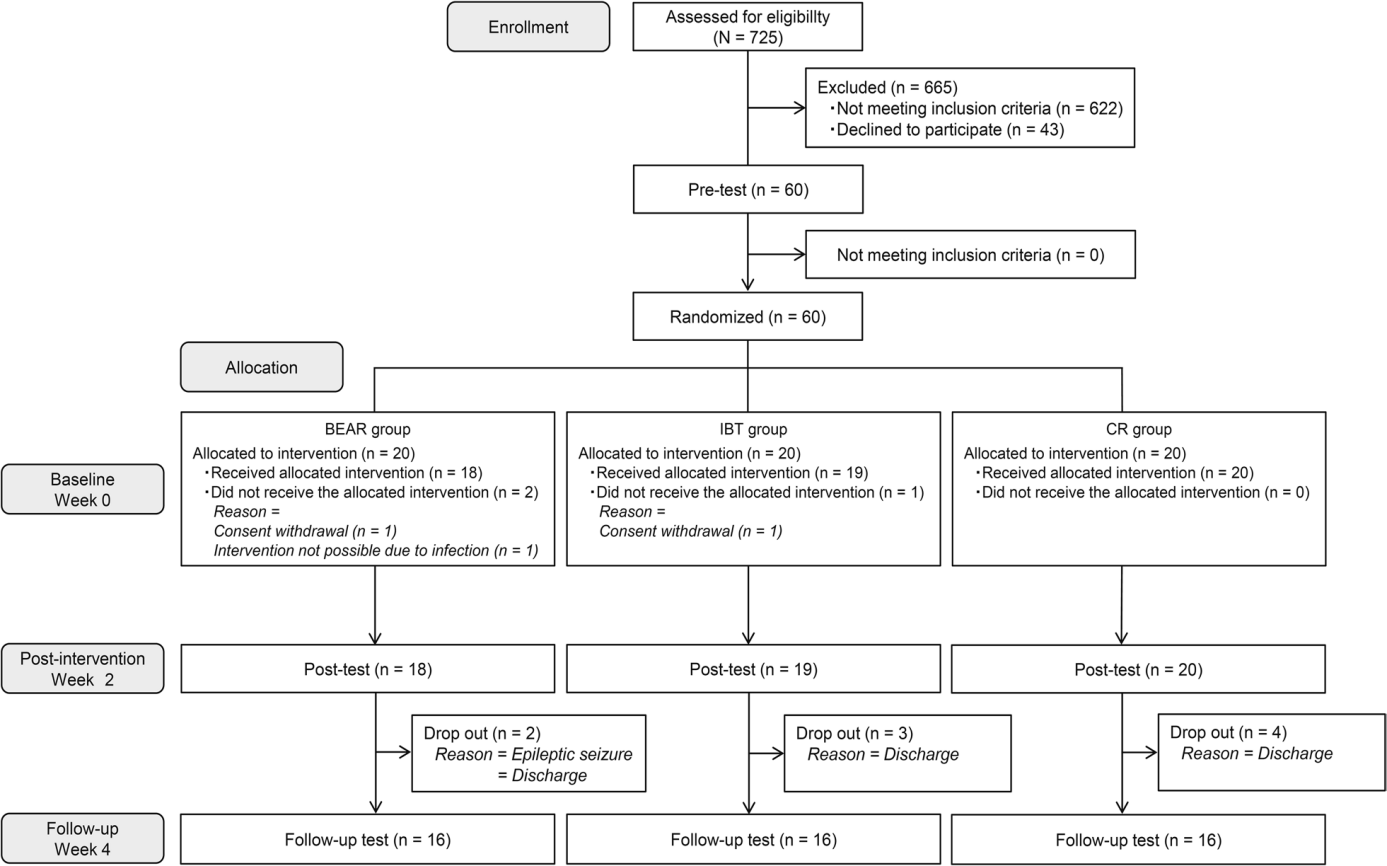
Flow diagram of participants

From December 2016 to February 2019, 725 consecutive patients were screened. Sixty patients who met the inclusion criteria were randomly allocated to one of the three groups (20 patients in each group). Fifty-seven patients completed the intervention, and 48 patients were evaluated at follow-up. Data concerning 57 patients, who completed the intervention, were analyzed (Fig. 3).

### Baseline data

Baseline comparisons of the patients included in the analyses are shown in Table 1. None of the variables, excluding age and sex, were significantly different among the groups. The mean (standard deviation [SD]) age of the patients was 64.9 (10.1) years, which was significantly different among the groups by ANOVA. The patients in the CR group were older than those in the other groups. Using Fisher’s exact test, statistically significant sex differences were found among the groups, with more male patients in the IBT group.

**Table 1:** Demographic and clinical characteristics of the patients at baseline

Characteristics		BEAR group	IBT group	CR group	*P*-value
		(n = 18)	(n = 19)	(n = 20)	
Sex, male/female, n		9/9	16/3	12/8	0.012
Age, years, mean (SD)		61.6 (10.1)	63.1 (10.1)	69.7 (8.7)	0.028
Height, cm, mean (SD)		162.4 (8.7)	165.9 (8.3)	161.6 (11.6)	0.320
Weight, kg, mean (SD)		61.3 (10.7)	62.8 (12.8)	60.1 (13.6)	0.794
Stroke type, hemorrhage/infarction, n		7/11	12/7	11/9	0.303
Affected side, right/left, n		13/5	6/13	9/11	0.052
Days from stroke onset, mean (SD)		55.8 (18.9)	58.2 (20.1)	52.2 (18.3)	0.615
Mini-BESTest, median (IQR)		9.5 (12.0)	14.0 (13.0)	15.0 (10.5)	0.883
SIAS lower limb motor score, median (IQR)		11.0 (3.0)	10.0 (3.0)	12.0 (3.0)	0.147
Maximum center of pressure movement, cm, mean (SD)					
	Left–right	31.2 (7.8)	32.0 (7.1)	33.3 (6.2)	0.659
	Anterior-to-posterior	12.9 (2.9)	12.6 (2.3)	13.3 (3.7)	0.775
Lower limb extension torque, Nm/kg, mean (SD)					
	Affected	0.7 (0.3)	0.7 (0.5)	0.8 (0.5)	0.998
	Non-affected	1.2 (0.4)	1.1 (0.5)	1.1 (0.4)	0.760
Lower limb muscle strength, N, mean (SD)					
Hip flexion	Affected	110.3 (76.1)	96.4 (88.5)	99.3 (66.8)	0.848
	Non-affected	162.2 (81.6)	154.6 (77.5)	137.0 (54.6)	0.541
Hip abduction	Affected	118.7 (59.4)	100.0 (59.4)	101.3 (60.1)	0.572
	Non-affected	138.6 (56.9)	141.8 (52.5)	134.3 (57.2)	0.916
Knee extension	Affected	135.7 (99.0)	117.9 (69.5)	144.4 (98.2)	0.647
	Non-affected	228.3 (112.0)	181.0 (82.2)	215.0 (95.3)	0.314
Knee flexion	Affected	71.9 (43.0)	72.8 (55.4)	78.7 (61.5)	0.914
	Non-affected	130.0 (40.2)	130.4 (45.2)	115.3 (53.4)	0.531
Ankle plantarflexion	Affected	116.9 (85.0)	80.7 (63.5)	99.6 (82.0)	0.370
	Non-affected	176.9 (85.4)	161.0 (95.9)	141.7 (82.3)	0.470
Ankle dorsiflexion	Affected	63.3 (44.6)	68.4 (63.3)	61.8 (45.5)	0.917
	Non-affected	98.2 (38.2)	109.6 (52.8)	90.3 (36.1)	0.380
Timed up and go test, sec, mean (SD)		18.0 (13.0)	19.5 (14.6)	17.4 (15.0)	0.895
Functional Ambulation Category, median (IQR)		3 (2.0)	3 (2.0)	4 (1.0)	0.642
Falls Efficacy Scale-International, median (IQR)		26.0 (17.0)	26.0 (20.0)	22.0 (11.0)	0.250
FIM, motor score, median (IQR)		67.0 (24.0)	68.0 (21.0)	74.0 (14.5)	0.415
FIM, cognition score, median (IQR)		31.5 (8.0)	32.0 (9.0)	28.5 (10.5)	0.926
FIM, total score, median (IQR)		98.5 (29.0)	97.0 (25.0)	104.0 (19.0)	0.548

*BEAR* Balance Exercise Assist Robot; *CR* conventional rehabilitation; *FIM* Functional Independence Measure; *IBT* intensive balance training; *IQR* interquartile range; *Mini-BESTest* Mini-Balance Evaluation Systems Test; *SD* standard deviation; *sec* seconds; *SIAS* Stroke Impairment Assessment Set

### Primary outcome

**Fig. 4:**
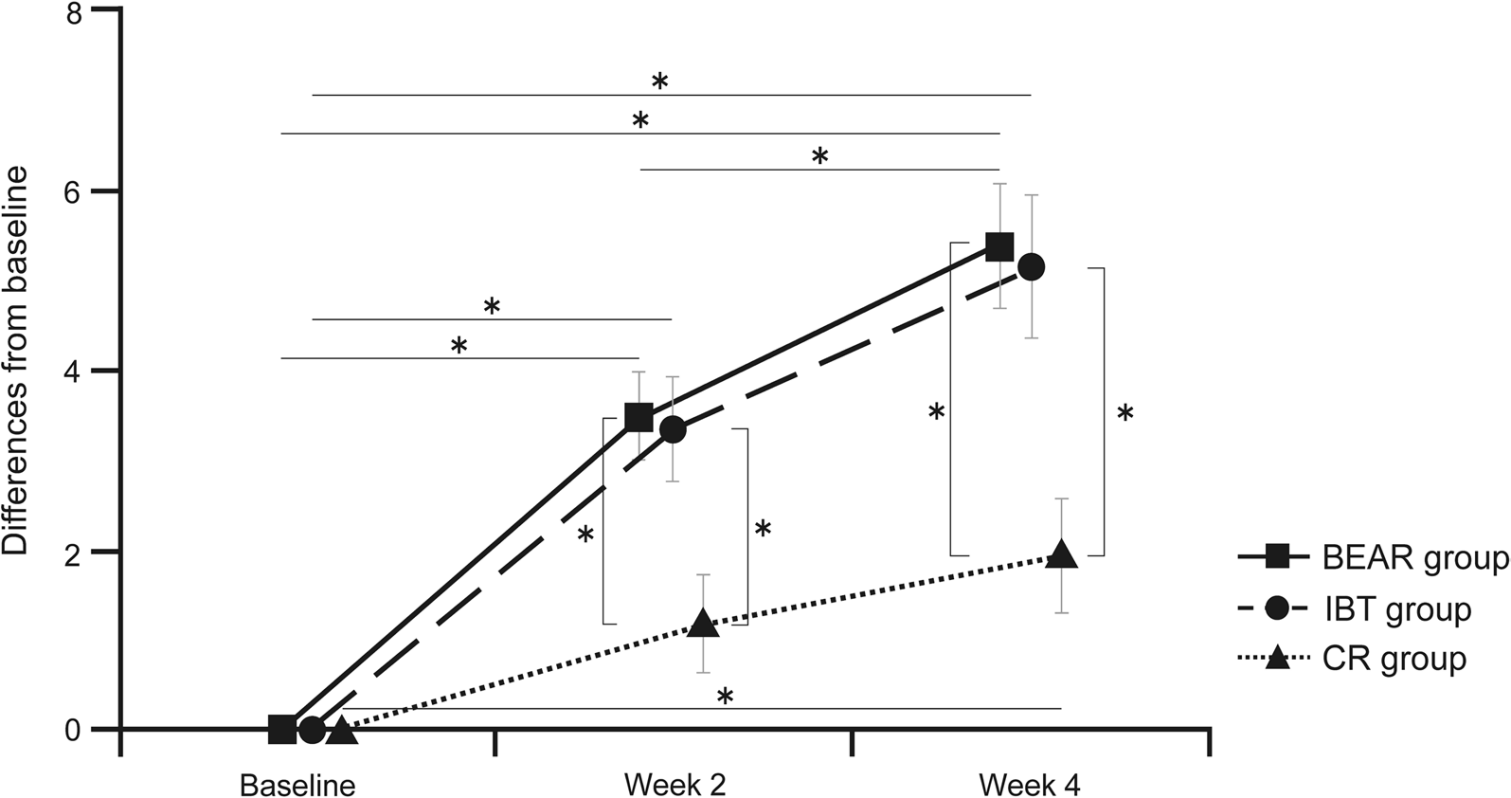
Over-time changes in the Mini-Balance Evaluation Systems Test scores among the groups. Error bars indicate standard errors. *Statistically significant between-group difference (P < 0.05). Bonferroni correction was used for multiple comparisons

Table 2 presents the total Mini-BESTest scores and subsystem changes in each group. Figure 4 shows the over-time changes in the total Mini-BESTest scores for each group.

**Table 2:** Changes in the Mini-Balance Evaluation Systems Test among the groups

		Change from baseline		Post-intervention (Week 2)					Follow-up (Week 4)				
		Post-intervention	Follow-up	Group effect			Post-hoc test*		Group effect			Post-hoc test*	
		Week 2	Week 4	*F*-value	*P*-value		vs. IBT	vs. CR	*F*-value	*P*-value		vs. IBT	vs. CR
Total score, mean (SD)	BEAR	3.5 (2.1) ^†^	5.4 (2.8) ^†‡^	6.90	0.003	BEAR	0.999	0.016	6.49	0.004	BEAR	0.999	0.006
	IBT	3.4 (2.5) ^†^	5.2 (3.1) ^‡^			IBT		0.003			IBT		0.012
	CR	1.2 (2.4)	1.9 (2.5) ^‡^			CR					CR		
Anticipatory, mean (SD)	BEAR	0.5 (0.9)	1.0 (1.0)^†^	0.65	0.525	BEAR			3.31	0.046	BEAR	0.999	0.050
	IBT	0.4 (0.8)	0.8 (0.9)^†^			IBT					IBT		0.195
	CR	0.0 (1.2)	0.2 (1.2)			CR					CR		
Reactive postural control, mean (SD)	BEAR	0.9 (1.3)	1.3 (1.5)^†‡^	1.10	0.342	BEAR			3.48	0.040	BEAR	0.803	0.035
	IBT	0.4 (1.3)	0.5 (1.3)			IBT					IBT		0.412
	CR	0.2 (1.2)	0.1 (0.9)			CR					CR		
Sensory orientation, mean (SD)	BEAR	0.4 (0.6)	0.8 (1.1)^†^	0.32	0.731	BEAR			0.29	0.751	BEAR		
	IBT	0.5 (0.7)	1.0 (1.2)^†^			IBT					IBT		
	CR	0.5 (0.8)^†^	0.8 (0.8)^†^			CR					CR		
Dynamic gait, mean (SD)	BEAR	1.7 (1.9)^†^	2.3 (1.5)^†^	5.94	0.005	BEAR	0.999	0.035	4.55	0.016	BEAR	0.999	0.041
	IBT	2.2 (2.1)^†^	2.9 (2.5)^†^			IBT		0.006			IBT		0.026
	CR	− 0.2 (1.8)	0.1 (1.8)			CR					CR		

*BEAR* Balance Exercise Assist Robot; *CR* conventional rehabilitation; *IBT* intensive balance training; *Mini-BESTest* Mini-Balance Evaluation Systems Test; *SD* standard deviation

Significant within-group difference from baseline^†^ and at 2 weeks^‡^. *When statistically significant between-group differences were found (P < 0.05), multiple comparisons between all groups were performed using the Bonferroni correction method

Post-intervention, the mean (SD) score changes from baseline in the BEAR, IBT, and CR groups were 3.5 (2.1), 3.4 (2.5), and 1.2 (2.4), respectively; these values were 5.4 (2.8), 5.2 (3.1), and 1.9 (2.5), at follow-up, respectively. In the ANCOVA using baseline values with age and sex as covariates, there was a significant group effect at post-intervention and at follow-up. The post-hoc test results indicated that the score changes in the BEAR and IBT groups were significantly greater than those in the CR group at post-intervention and follow-up. No significant differences were found between the BEAR and IBT groups. Regarding within-group changes in the Mini-BESTest, a significant improvement was found in all groups between baseline and follow-up. Moreover, a significant improvement was recorded between baseline and post-intervention in the BEAR and IBT groups. A significant improvement was found from post-intervention to follow-up only in the BEAR group.

Score changes in the Mini-BESTest in each system are shown in Table 2. Significant group effects were recorded in dynamic gait at post-intervention and in all systems, except for sensory orientation at follow-up (*P* < 0.05). In the pairwise comparisons, the score changes of dynamic gait were greater in the IBT and BEAR groups than in the CR group at post-intervention and follow-up (*P* < 0.05). Regarding reactive postural control, the BEAR group showed a significant improvement compared with the CR group at follow-up (*P* = 0.035).

### Secondary outcomes

The results of secondary outcomes are presented in Tables 3 and 4. Except for the post-intervention assessment of the Timed Up and Go test, there were no between-group effects for any measures, either post-intervention or follow-up. The mean (SD) change in the Timed Up and Go test scores from baseline to the post-intervention assessment for each group was − 4.2 (6.0), − 2.2 (3.5), and − 0.9 (2.3) in the BEAR, IBT, and CR groups, respectively. On the ANCOVA using baseline values with age and sex as covariates, there was a significant group effect post-intervention. The post-hoc test results indicated that the BEAR group showed a significant improvement compared to the CR group (*P* = 0.023).

**Table 3:** Changes in muscle strength among the groups

		Change from baseline				Post-intervention (Week 2)		Follow-up (Week 4)	
		Post-intervention		Follow-up		Group effect		Group effect	
		(Week 2)		(Week 4)		*F*-value	*P*-value	*F*-value	*P*-value
Affected side									
Lower limb extension torque, Nm/kg, mean (SD)	BEAR	0.2	(0.2)^†^	0.2	(0.1)^†^	0.13	0.878	0.14	0.873
	IBT	0.2	(0.6)	0.1	(0.2)				
	CR	0.1	(0.2)^†^	0.1	(0.2)^†^				
Hip flexion, N, mean (SD)	BEAR	19.7	(35.6)	3.3	(33.9)	2.36	0.105	0.17	0.840
	IBT	− 6.2	(71.9)	19.2	(25.1)				
	CR	0.9	(30.1)	− 3.3	(44.6)				
Hip abduction, N, mean (SD)	BEAR	13.6	(35.1)	4.9	(36.8)	0.82	0.444	0.04	0.964
	IBT	2.8	(37.3)	8.1	(31.3)				
	CR	− 0.7	(24.5)	3.7	(24.3)				
Knee extension, N, mean (SD)	BEAR	21.0	(52.4)	24.0	(45.0)	2.29	0.112	0.29	0.751
	IBT	14.4	(44.9)	13.6	(41.0)				
	CR	− 24.4	(55.3)	9.6	(63.3)				
Knee flexion, N, mean (SD)	BEAR	26.0	(56.9)	6.1	(35.1)	0.86	0.429	0.80	0.457
	IBT	5.9	(40.0)	2.1	(18.2)				
	CR	4.7	(17.4)	5.8	(20.8)				
Ankle plantarflexion, N, mean (SD)	BEAR	6.6	(63.0)	− 6.7	(54.1)	0.00	0.999	2.21	0.122
	IBT	26.5	(43.6)	47.1	(67.9)^†^				
	CR	6.8	(78.8)	6.2	(58.8)				
Ankle dorsiflexion, N, mean (SD)	BEAR	6.5	(33.0)	11.8	(38.6)	0.42	0.661	0.41	0.666
	IBT	9.1	(27.4)	10.1	(13.2)				
	CR	− 1.0	(27.6)	3.7	(25.0)				
Non-affected side									
Lower limb extension torque, Nm/kg, mean (SD)	BEAR	0.1	(0.2)	0.1	(0.1)^†^	0.65	0.529	1.14	0.328
	IBT	0.1	(0.2)	0.1	(0.2)				
	CR	0.1	(0.1)	0.3	(0.6)				
Hip flexion, N, mean (SD)	BEAR	− 3.3	(74.6)	− 23.8	(72.7)	0.46	0.635	1.25	0.297
	IBT	− 5.1	(68.5)	1.6	(27.7)				
	CR	− 12.6	(33.3)	− 2.1	(40.4)				
Hip abduction, N, mean (SD)	BEAR	22.4	(42.1)	− 3.2	(41.2)	1.23	0.300	0.51	0.604
	IBT	12.9	(41.1)	6.4	(38.5)				
	CR	− 6.8	(37.8)	− 1.7	(28.6)				
Knee extension, N, mean (SD)	BEAR	− 7.3	(73.3)	2.7	(64.3)	2.94	0.062	0.07	0.928
	IBT	21.4	(62.9)	9.7	(67.1)				
	CR	− 42.3	(59.8)^†^	− 2.3	(47.7)^‡^				
Knee flexion, N, mean (SD)	BEAR	11.5	(32.2)	10.3	(32.6)	2.02	0.143	0.85	0.434
	IBT	− 6.8	(37.0)	10.3	(37.2)				
	CR	− 9.2	(24.4)	10.3	(25.0)^‡^				
Ankle plantarflexion, N, mean (SD)	BEAR	− 3.5	(75.3)	− 8.4	(64.5)	0.79	0.458	1.01	0.371
	IBT	36.0	(59.8)	21.6	(82.5)				
	CR	3.8	(70.5)	20.1	(58.3)				
Ankle dorsiflexion, N, mean (SD)	BEAR	13.7	(35.3)	2.9	(28.6)	0.84	0.439	0.95	0.393
	IBT	1.0	(40.2)	16.6	(29.6)				
	CR	− 5.0	(35.0)	− 3.0	(32.9)				

*BEAR* Balance Exercise Assist Robot; *CR* conventional rehabilitation; *IBT* intensive balance training; *SD* standard deviation

Significant within-group difference from the baseline^†^ and from 2 weeks^‡^

**Table 4:** Changes in other secondary outcomes among the groups

		Change from baseline				Post-intervention (Week 2)				Follow-up (Week 4)	
		Post-intervention		Follow-up		Group effect		Post-hoc test *		Group effect	
		(Week 2)		(Week 4)		*F*-value	*P*-value	vs. IBT	vs CR	*F*-value	*P*-value
SIAS lower limb motor score, mean (SD)	BEAR	0.9	(1.4)^†^	0.8	(1.4)	0.46	0.634			0.09	0.912
	IBT	0.6	(1.9)	0.9	(2.0)						
	CR	0.1	(0.9)	0.3	(1.1)						
Maximum COP movement, left–right, cm, mean (SD)	BEAR	3.2	(5.7)^†^	3.2	(5.2)^†^	0.62	0.543			0.19	0.825
	IBT	1.9	(4.7)	2.2	(4.6)						
	CR	0.1	(5.7)	1.4	(6.4)						
Maximum COP movement, anterior-to-posterior, cm, mean (SD)	BEAR	0.7	(2.6)	1.1	(2.6)	0.12	0.889			0.04	0.963
	IBT	0.9	(2.4)	1.1	(3.0)						
	CR	0.1	(3.6)	0.7	(3.5)						
Functional Ambulation Category, mean (SD)	BEAR	0.7	(0.8)^†^	0.7	(0.6)^†^	0.47	0.629			2.20	0.123
	IBT	0.5	(0.6)^†^	0.8	(0.7)^†^						
	CR	0.4	(0.6)^†^	0.9	(0.8)^†‡^						
Timed Up and Go test, sec, mean (SD)	BEAR	− 4.2	(6.0)^†^	− 4.5	(6.5)^†^	4.07	0.023	0.216	0.022	1.84	0.171
	IBT	− 2.2	(3.5)^†^	− 2.6	(3.8)^†^				0.999		
	CR	− 0.9	(2.3)	− 1.7	(3.7)						
Falls Efficacy Scale-International, mean (SD)	BEAR	− 0.9	(10.2)	− 3.8	(7.1)	0.46	0.636			0.12	0.890
	IBT	− 3.4	(9.5)	− 4.1	(8.5)						
	CR	− 0.7	(5.2)	− 2.4	(6.2)						
FIM, motor score, mean (SD)	BEAR	9.8	(8.6)^†^	13.4	(9.3)^†^	2.07	0.136			1.71	0.193
	IBT	9.4	(7.8)^†^	9.3	(9.2)^†^						
	CR	3.6	(3.9)^†^	9.2	(6.7)^†‡^						
FIM, cognition score, mean (SD)	BEAR	1.1	(2.3)	1.1	(2.1)^†^	0.34	0.717			0.52	0.598
	IBT	0.9	(2.2)	1.3	(2.9)^†^						
	CR	0.6	(1.3)	1.1	(1.9)^†^						
FIM, total score, mean (SD)	BEAR	10.9	(9.4)^†^	14.5	(9.3)^†^	2.37	0.104			1.82	0.174
	IBT	10.3	(8.2)^†^	15.0	(11.2)^†^						
	CR	4.2	(4.7)^†^	10.3	(7.4)^†‡^						

*BEAR* Balance Exercise Assist Robot; *COP* center of pressure; *CR* conventional rehabilitation; *FIM* Functional Independence Measure; *IBT* intensive balance training; *SD* standard deviation; *SIAS* Stroke Impairment Assessment Set

Significant within-group difference from baseline^†^ and from 2 weeks^‡^. *When statistically significant between-group differences were found (P < 0.05), multiple comparisons were performed between all the groups using the Bonferroni correction method

**Fig. 5:**
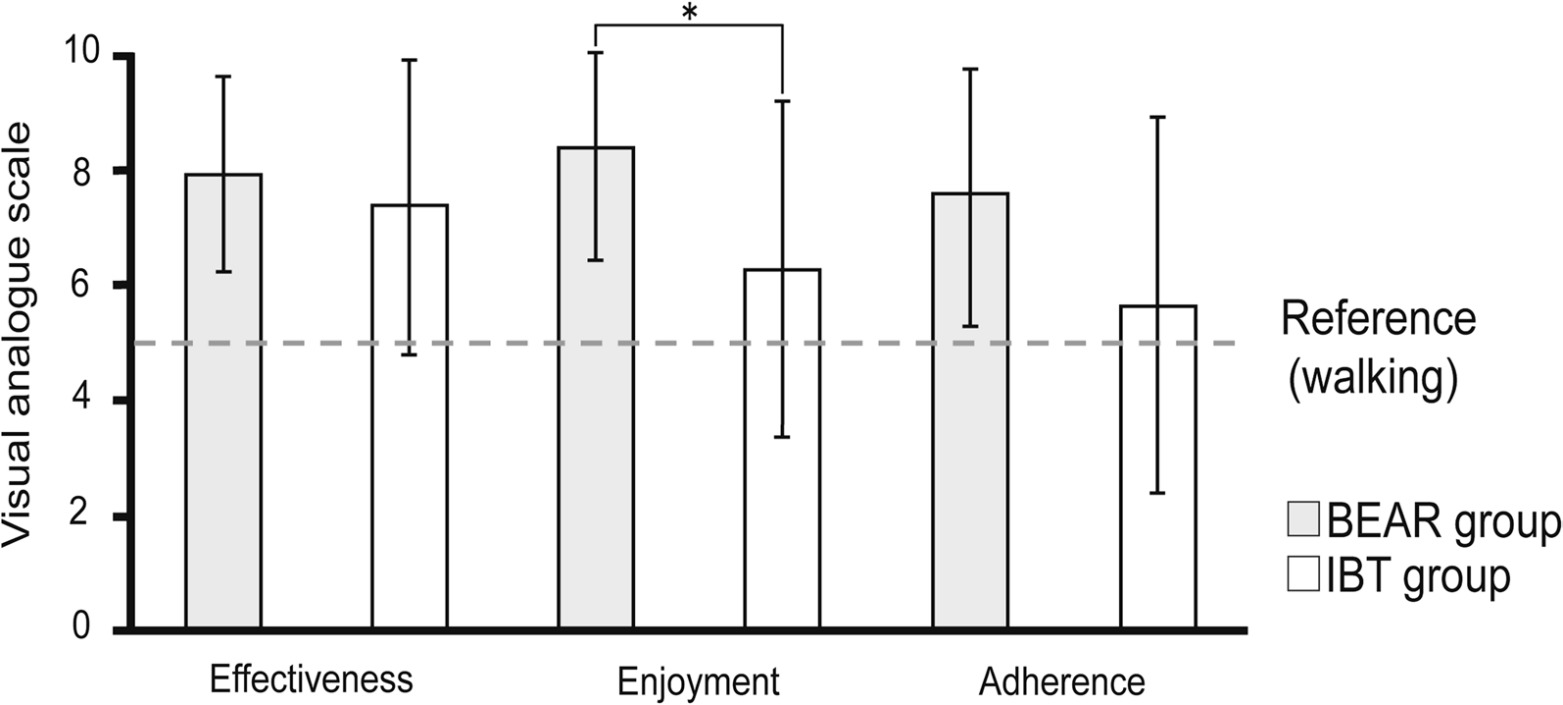
Questionnaire results following the interventions. Regular walking training was used as a reference (score 5). *Statistically significant difference (P < 0.05)

The results of the questionnaires concerning the BEAR and IBT groups are shown in Fig. 5. The mean (SD) scores for each question in the BEAR and IBT groups were 7.9 (1.7) and 7.3 (2.6), respectively, for effectiveness; 8.4 (2.0) and 6.3 (2.9), respectively, for enjoyment; and 7.6 (2.2) and 5.7 (3.3), respectively, for adherence. While no significant between-group difference was found in “Effectiveness” (*P* = 0.688), the patients in the BEAR group showed significantly greater scores than those in the IBT group in “Enjoyment” (*P* = 0.020) and a marginally significant difference in “Adherence” (*P* = 0.080).

One patient had a fall in the BEAR group, there were four falls among three patients in the IBT group, and two patients had three falls in the CR groups. All the falls occurred outside the training sessions. There was no significant difference in the number of falls between the groups (*P* = 0.456).

No adverse events directly due to intervention were observed throughout the study. In the BEAR group, one patient had an epileptic seizure, and one patient was infected with scabies outside the training session.

## Discussion

We aimed to determine whether balance-focused robotic training in combination with conventional inpatient rehabilitation facilitated balance improvement in patients with subacute hemiparetic stroke. Post-intervention, the BEAR and ITB groups displayed significantly improved dynamic balance compared to that of the CR group, including an improved follow-up exceeding 4 points, which was previously reported as a minimal clinically important difference [29]. Furthermore, the results from the questionnaire revealed that patients in the BEAR group enjoyed the training more than those receiving supervised IBT.

Notably, a significant balance improvement was observed in the CR group, suggesting that an apparent sufficient training effect was produced by conventional rehabilitation alone. Nevertheless, patients in both the BEAR and IBT groups, with additional balance training, demonstrated significantly improved balance compared to the CR group. This additive effect is likely due to the increased training they received, which specifically targeted balance function. Generally, repeating task-specific training is important for improving specific functions [6]. Our findings supported this, as only performance in the Mini-BESTest and Timed Up and Go test improved in intervention groups, compared to the CR group, whereas muscle strength and activities of daily living did not. Importantly, balance can be subdivided into several components [2], and the Mini-BESTest separately evaluates these components for dynamic balance. Notably, only the BEAR group showed a significant improvement in reactive postural control. The difference between the tasks included in the IBT and BEAR groups is that both groups include anticipatory postural control tasks, whereas the BEAR group includes a reactive postural balance task (rodeo task). Therefore, this was also a task-specific effect. Since tasks focused on reactive postural control are difficult to conduct with supervised training by a therapist, a great advantage of robotic balance training is that it can produce irregular disturbances that cannot be produced by humans. This finding is consistent with that of a previous study [46] reporting that perturbation-based balance training with a movable platform improved reactive steps in patients with chronic stroke.

Most robots for lower extremities used in stroke rehabilitation are intended for gait improvement [7]; few technology-assisted training devices have been proposed specifically to improve the balance function. In one study involving Hunova (Movendo Technology Srl, Genoa, IT), a balance robot consisting of two servo-controlled platforms for the feet and a seat in which one performs passive and active exercises in the sitting and standing positions, patients with chronic stroke trained for 45 min three times a week for 5 weeks and reported improvements in balance function, although no difference was found compared with dose-matched conventional balance training [47]. Another balance robot consisting of a standing balance training apparatus, namely, the BalanceReTrainer, is a mechanical apparatus that provides a safe balancing environment, wherein the balancing efforts of a standing individual are augmented through stabilizing forces acting at the level of the pelvis, assisting the patients in the balancing activity [48]. Using this device, one study found that a patient with chronic stroke and hemineglect improved weight shift on the affected leg after 20 min of training five times a week for 2 weeks [48]. One unique feature of the BEAR that sets it apart from the robots used in previous studies [47, 48] is that the BEAR dynamically moves in conjunction with slight changes in the center of pressure of the patients, enabling augmented feedback during postural control tasks, even for patients with limited balance ability. Furthermore, while previous studies have been limited to patients with chronic stroke [47, 48], our trial assessed the effects of a balance-specific robot on balance function in patients with subacute stroke. Importantly, improvement in balance function was obtained despite a relatively short intervention period (18 min), which was 50% shorter than that observed in a previous study [47]. Further studies are warranted to determine whether this benefit was attributable to the characteristics of the robot or the patients; however, this study clearly demonstrates the benefit of a robot specialized for balance training in patients with subacute stroke.

Motivation is an important factor in rehabilitation that has been reported to increase adherence to exercise [49]. Enjoyment contributes to internal motivation by encouraging individuals to perform the activity and to remain motivated [50]. Importantly, game elements enhance balance training enjoyment, even among older individuals, and the decrease in enjoyment over time tends to be less pronounced in gamified exercises than in conventional balance training [51]. In this study, the patients in the BEAR group enjoyed training significantly more than those in the IBT group, and there was a trend toward higher adherence. This is likely because the BEAR also included gaming features that helped patients to focus more on balance training, such as the ability to control their body movements on a monitor synchronized with the robot. This suggests that balance training using the BEAR helped to maintain patients’ motivation, thus, contributing to a positive effect.

No direct adverse events were observed during training using the BEAR, which further supports the clinical applicability of the device. Furthermore, training using the BEAR does not require the presence of a rehabilitation professional, unlike IBT, which requires professional instructions and guidance. However, for safety, a non-professional person is required during BEAR training even though it has a safety harness. There is also a substantial cost for installing the device. Considering that the patients’ impressions of the training were better in the BEAR group than in the IBT group, the effectiveness of BEAR training may be superior to IBT with a longer intervention period. However, in terms of cost-effectiveness, the superiority of the BEAR or IBT cannot be conclusively proven based on our findings. Modifications to improve the safety of the BEAR, such as using a footplate placed lower than the current footplate level, would preclude the requirement for a human assistant and make the BEAR more advantageous than its current form.

This study has some limitations. First, this study was conducted at a single facility, involving patients with stroke in the subacute phase. Thus, generalizing the findings to different settings and stroke populations in different phases should be considered with caution. Additionally, the follow-up period of this study, which was 2 weeks, was short. Future studies with longer follow-up periods are required to confirm the long-term effects of BEAR training. Furthermore, age and sex were found to be significantly different between the groups. Age and sex have been reported to be associated with postural control and balance performance [52, 53]; age also affects balance improvement by training [54]. The effects of the age- and sex-differences on the findings of the present study are unknown, and while we attempted to remove the influence of these difference on the results using statistical methods (ANCOVA), it is not known whether these effects were completely removed. Further, the results of the Mini-BESTest subscore analyses should be interpreted with caution, as the power may not have been adequate because the sample size was calculated based on the total Mini-BESTest score. It is possible that the other subsystems of the Mini-BESTest may also show statistically significant difference as the sample size increases; however, our findings, namely, the significant improvement in the dynamic gait system in the intervention group than in the CR group, and the significant improvement in the reactive postural control system in the BEAR group than in the CR group, are robust and reliable.

## Conclusions

The addition of robotic balance training using BEAR or supervised intensive balance training to conventional rehabilitation was effective in improving balance in patients with hemiparetic subacute stroke. Moreover, subjective enjoyment was greater in patients using the BEAR than in those undergoing supervised intensive balance training.

## Data Availability

The datasets used and/or analyzed during the current study are available from the corresponding author on reasonable request.

## Abbreviations

BEAR: Balance Exercise Assist Robot
CR: conventional inpatient rehabilitation
IBT: intensive balance training
Mini-BESTest: Mini-Balance Evaluation Systems Test
FIM: Functional Independence Measure
IQR: interquartile range
SD: standard deviation
sec: seconds
SIAS: Stroke Impairment Assessment Set
